# 3D MRI PD‐SPACE‐COR Predicting Safety Margin for Coracoid Transfer

**DOI:** 10.1111/os.13719

**Published:** 2023-04-24

**Authors:** Zhongkai Ren, Xiaohong Huang, Haining Peng, Jinlong Ma, Yingze Zhang, Tengbo Yu

**Affiliations:** ^1^ Department of Sports Medicine Affiliated Hospital of Qingdao University Qingdao China; ^2^ Shandong Institute of Traumatic Orthopedics, Medical Research Center The Affiliated Hospital of Qingdao University Qingdao China; ^3^ Department of Orthopedics Affiliated Hospital of Qingdao University Qingdao China; ^4^ Department of Orthopedics The Third Hospital of Hebei Medical University Shijiazhuang China; ^5^ Department of Orthopedic Surgery Qingdao Hospital, University of Health and Rehabilitation Sciences (Qingdao Municipal Hospital) Qingdao China

**Keywords:** 3D MRI, Cadaveric measurement, Coracoid transfer, Latarjet, PD‐SPACE‐COR, Safety margin

## Abstract

**Objective:**

The maximum bone length available for coracoid process transfer varies among individuals, while no preoperative guideline has been developed for predicting the safety margin (SM) in Latarjet shoulder reconstruction. The aim of the study was to evaluate the 3D MRI proton density (PD)‐weighted sampling perfection with application‐optimized contrasts using different flip‐angle evolution (SPACE) sequence in preoperatively predicting SM for coracoid transfer.

**Methods:**

The post‐multiplanar reconstructed images were obtained from 24 volunteers (17 males, seven females) to determine the clarity and sensitivity of the PD‐SPACE‐COR and PD‐SPACE‐FS‐COR protocols. Furthermore, the distance from the coracoid tip to the lateral edge of the attachment of trapezoid ligament (TL) was measured. To evaluate the accuracy of 3D MRI prediction, a cadaveric cohort has been launched in 32 shoulders (nine males, seven females). The distance between the tip of coracoid process and the outmost edge of TL footprint, namely, the SM, was measured.

**Results:**

A better sensitivity was found in PD‐SPACE‐COR in detecting coracoclavicular ligaments (CCLs), including TL and conoid ligament (CL), compared to PD‐SPACE‐FS‐COR by ranking, McNemar test (*P* = 0.001), and kappa coefficients (κ = 0.51, *P* = 0.43). The SM determined by the PD‐SPACE‐COR protocol was 24.28 ± 2.17 mm while that by cadaveric morphometry was 25.53 ± 2.84 mm. No difference was found between measurements (*P* = 0.78).

**Conclusion:**

This research provides new insights for preoperatively geometrical planning coracoid transfer by 3D MRI PD‐SPACE‐COR, which motivates personalized medicine in orthopedics.

## Graphical abstract

3D MRI PD‐SPACE‐COR is qualified for preoperatively predicting safety margin in coracoid transfer, which helps optimizing the surgical decision and may motivate the development of personalized medicine in the Latarjet shoulder reconstruction surgery.

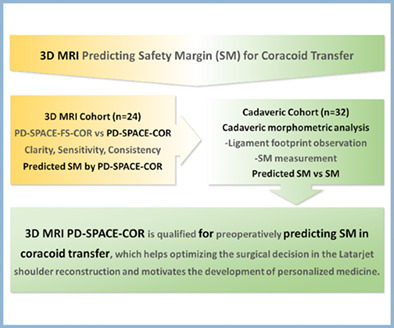

## Introduction

Latarjet coracoid process transfer is broadly performed in treating anterior shoulder instability based on the glenoid bone loss and coracoid process dimensions.^1^ The safety margin (SM) for coracoid process transfer, that is, the maximum coracoid bone length from coracoid tip for osteotomy, matters in optimizing surgical options for glenoid reconstruction. Moreover, the bone distance around the screw has been associated with tolerance of screw‐positioning error in Latarjet.^2^ Attributing to the minimum coracoid bone graft length for safe screw insertion and the maximum osteotomy length that varies among individuals, preoperatively predicting SM is advocated for an efficient shoulder surgical intervention.

Attempts have been made to determine the bony landmark for osteotomy in Latarjet coracoid transfer using CT,^3^ which overlooks the coracoclavicular ligaments (CCLs) and may cause coracoclavicular injury during osteotomy and comorbid diseases.^4^, ^5^ MRI combines an excellent soft‐tissue contrast and a comprehensive evaluation of the osseous structures, which makes it indispensable in orthopedics.^6^ 2D MRI has been frequently performed in musculoskeletal imaging.^7^ The most complete and clearest layer of the CCLs of the T2‐weighted MR images was selected to shape the CCLs and its bone attachment.^8^ However, similar outcomes were found between patients with or without preoperative 2D MRI evaluation 2 years after the Latarjet surgery.^9^ The 3D arthrography with great spatial resolution and multiplanar reconstruction (MPR) technique helps surgeon decisions in tendon disruptions and ligament lesions.^6^ Hence, the technique of 3D MRI has been reported in assessing the bone loss in the glenoid or humeral head.^10^, ^11^, ^12^ Moreover, 3D proton density (PD)‐weighted turbo spin‐echo (TSE) sampling perfection with application‐optimized contrasts using different flip‐angle evolution (SPACE) has been applied in intraarticular pathology detection of shoulder joints.^13^

The aims of this study were to: (i) assess the feasibility of 3D MRI PD‐weighted SPACE sequences in imaging the CCL attachments, including the PD‐SPACE‐COR and PD‐SPACE‐FS‐COR (fat‐suppressed) protocols; and (ii) determine the SM of the cadaveric cohort, and evaluate the reliability of PD‐SPACE‐COR in preoperatively predicting SM by comparing the 3D MRI measurement with the cadaveric morphometric data. With the potential to motivate personalized medicine, the outcomes of this study provide new insights for preoperatively geometrical planning in Latarjet shoulder reconstruction.

## Materials and Methods

### 3D MRI PD‐SPACE Measurement

#### Participants and Parameters

The 3D MRI was performed on 24 volunteers (10 left shoulders, 14 right shoulders; 17 males, seven females; aged 30.7 ± 8.99, range 25–57; height 176.21 ± 5.64 cm) free from shoulder trauma history or participation in heavy manual labor of upper limbs. Both PD‐SPACE‐COR and PD‐SPACE‐FS‐COR protocols (Table 1) under 3D MRI PD‐SPACE sequence have been launched on a 3.0‐T MRI scanner (MAGNETOM Skyra, Siemens Healthcare, Erlangen, Germany). All volunteers were asked to maintain a supine position with their arms neutrally on the body side. Images were acquired in coronal oblique plane (parallel to the supraspinatus tendon) and reformatted in transversal and sagittal oblique planes. Informed consents have been obtained from all volunteers in the 3D MRI cohort and families in the cadaveric cohort. The protocol of this study has been approved by the institutional review board of The Affiliated Hospital of Qingdao University (No. QYFYWZLL26070).

**TABLE 1:** The parameters of PD‐SPACE‐COR and PD‐SPACE‐FS‐COR

Parameter	PD‐SPACE‐COR	PD‐SPACE‐FS‐COR
TR/TE (ms)	1100/37	1100/37
FOV (mm)	180	180
Matrix	320 × 320	320 × 320
Slice thickness (mm)	0.9	0.9
Slice gap (mm)	0	0
Acquisition frequency	1.4	1.4
Acquisition time (s)	7:05	7:25

Abbreviations: COR: coronal; FOV: Field‐of‐view; FS: fat‐suppressed; PD: proton density weighted; SPACE: 3D sampling perfection with application‐optimized contrasts using different flip‐angle evolution; TE: echo time; TR: repetition time.

#### Structural Outcome Measures

The clarity of the attachments of the CCLs on the coracoid process in the MRI, including trapezoid ligament (TL) and conoid ligament (CL), were taken as the criterion in evaluating the sensitivity of PD‐SPACE‐COR and PD‐SPACE‐FS‐COR protocols in CCL imaging. One radiologist (J.M.) and two orthopedic surgeons (Z.R. and H.P.) independently ranked the clarity of images from two protocols. The sensitivity was represented by percentage (%).

**Fig. 1:**
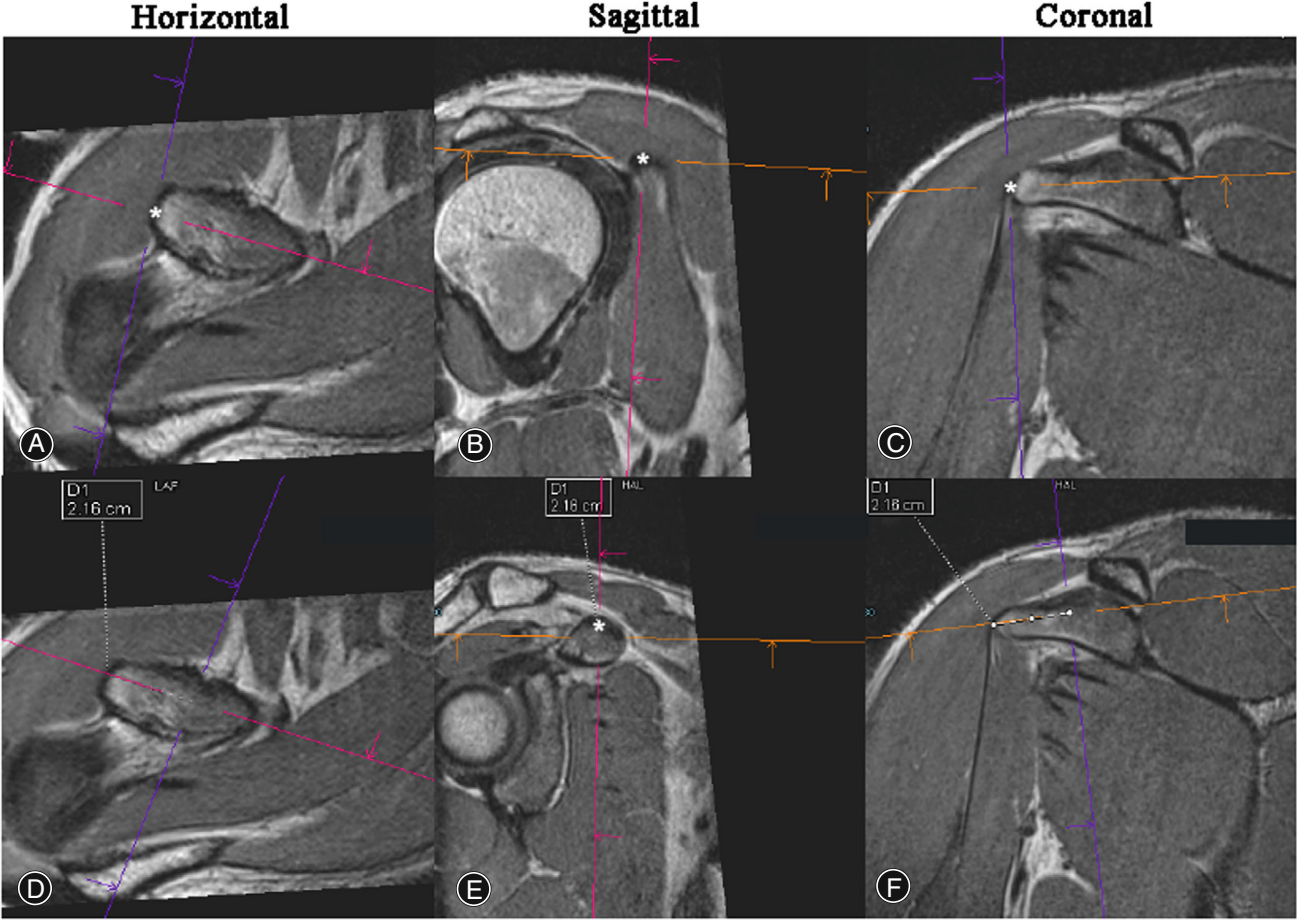
Data acquisition from 3D MRI PD‐SPACE‐COR imaging. The coracoid tip is identified as the point most distant from the base on the coracoid on the horizontal plane. The tip of the coracoid process has been localized with an asterisk in the horizontal (A), Sagittal (B), Coronal (C) planes. Meanwhile, the lateral edge of the attachment of trapezoid ligament (TL) has been localized with an asterisk in the horizontal plane (D) and sagittal plane (E). and, the predicted SM, namely, the distance between two asterisks (represent the coracoid tip and lateral edge of TL attachment) was measured (F).

With the post‐MPR images obtained from the 3D MRI PD‐SPACE‐COR protocol, the distance between the coracoid tip and lateral edge of the attachment of TL was identified as the predicted SM (Fig. 1) and independently measured post MPR in the workstation (Syngo. *via*, Siemens, Erlangen, Germany; measurement accuracy: ± 0.1 mm) by three researchers (Z.R., H.P., and J.M.). The data was expressed as mean ± standard deviation (range).

### Cadaveric Morphometric Analysis

#### Specimen Preparation

**Fig. 2:**
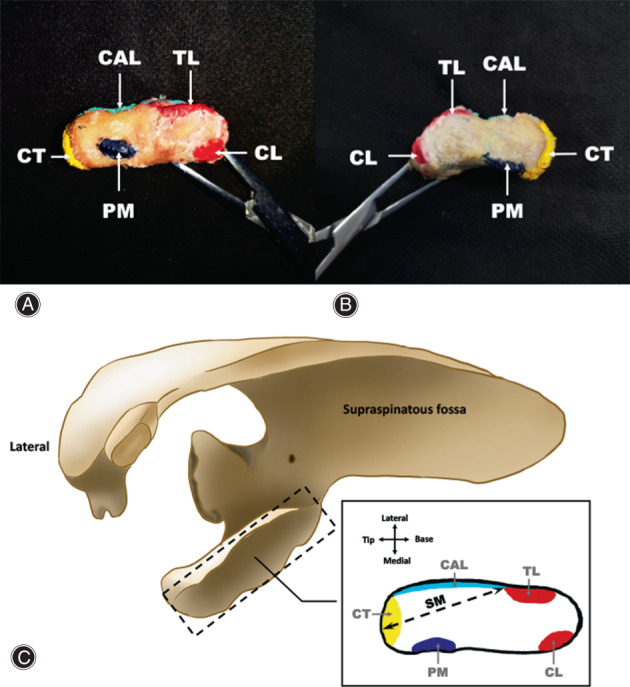
The ligament footprints on the surface of coracoid process. (A). indicates the top view of the left coracoid process, and (B). indicates the top view of the right coracoid process. The locations of the ligament footprints on the coracoid process, including the conjoint tendon (CT, yellow), coracoacromial ligament (CAL, light blue), pectoralis minor (PM, blue), trapezoid ligament (TL, red), and conoid ligament (CL, red), have been indicated in (C). The SM was identified as the tip of the coracoid process to the outmost edge of TL footprint on the specimen.

Tactile measurement was performed on 32 human cadaveric shoulders (16 left, 16 right; nine males, seven females) with no prior injury, surgical history, and anatomical abnormalities. The specimens were fixed with 37% formaldehyde solution, and all soft tissues (skin, subcutaneous tissues, deltoid, and pectoralis major muscle) were removed to expose the coracoid process, conjoint tendon (CT), coracoacromial ligament (CAL), pectoralis minor (PM), and CCLs. The root of the coracoid process was sawn off, ligaments and tendons were removed from the coracoid process (Fig. S1). Subsequently, the footprints of tendons and ligaments were pigmented for observation and morphometry (Fig. 2(A),(B)).

#### Data Acquisition

The SM in the specimens was identified as the distance between the tip of coracoid process and the outmost edge of TL footprint (Fig. 2(C)). The SM was measured with an electronic digital vernier caliper (111‐101, Sanliang, Dongguan, China; measurement accuracy: ± 0.01 mm) for three times, and the distance was audio‐recorded for subsequent analysis. The data was expressed as mean ± standard deviation (range).

### Statistical Analysis

SPSS 25.0 (IBM, Armonk, NY) was employed. The McNemar and Cohen's kappa tests were recruited to determine the consistency between the sensitivities of two protocols. After the homogeneity of variance was determined with Levene's test, the outcomes measured by the PD‐SPACE‐COR protocol and cadaveric morphometry were compared using chi‐squared test. Moreover, the gender difference was determined using chi‐squared test. The *P*‐values were not adjusted for multiple testing, and their interpretation was explorative. A value of *P* < 0.05 was considered statistically significant.

## Results

### PD‐SPACE‐COR Precedes PD‐SPACE‐FS‐COR in Detecting Safety Margin of Coracoid Process

**Fig. 3:**
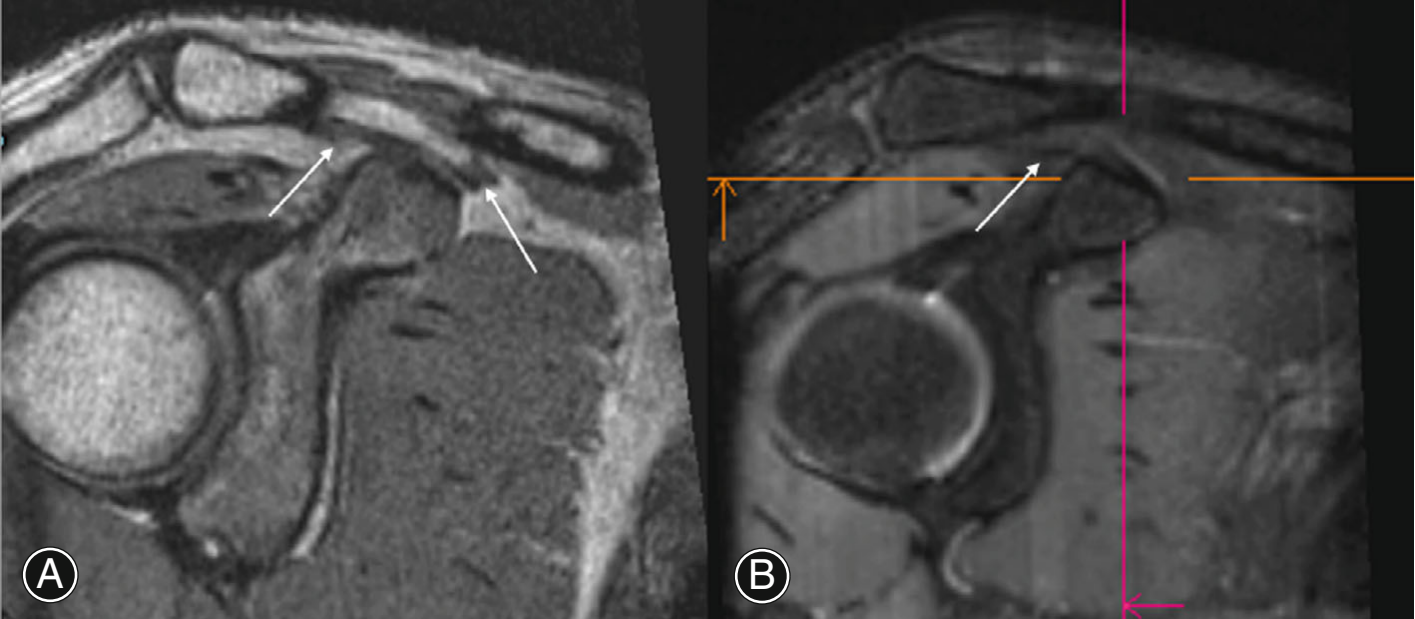
The representative images from (A) PD‐SPACE‐COR and (B) PD‐SPACE‐FS‐COR post‐MPR. The clarity of the attachments of trapezoid ligament (TL, left arrow in A) and conoid ligament (CL, right arrow in A) is good in PD‐SPACE‐COR imaging while that of TL (arrow in B) is not distinct and CL attachment is absent by PD‐SPACE‐FS‐COR imaging.

The clarity of the attachments of TL and CL is good in PD‐SPACE‐COR imaging, while that of TL is not vague and CL is absent in PD‐SPACE‐FS‐COR imaging (Fig. 3). Also, the sensitivity of CCLs was 95.8% for PD‐SPACE‐COR and 37.5% for PD‐SPACE‐FS‐COR. The inconsistency between PD‐SPACE‐COR and PD‐SPCAE‐FS‐COR was determined by the McNemar test (*P* < 0.001) and Cohen's kappa test (κ = 0.51, *P* = 0.43).

### The Safety Margin Is Determined Based on the Ligament Footprints on the Coracoid Process

As it has been shown in Fig. 2(C), the footprints of the CAL and TL were conjunct and located on the lateral side of the coracoid process with CAL closed to the tip and TL closed to the base of the coracoid process. Moreover, the PM and CL were located on the medial aspect of the coracoid process.

### The PD‐SPACE‐COR Assessment Is Consistent with the Cadaveric Morphometric Outcome

As it has been shown in Table 2, the maximum bone length available for osteotomy in coracoid process transfer was 24.28 ± 2.17 mm predicted by 3D MRI PD‐SPACE‐COR, and the average maximum bone length obtained from the cadaveric cohort was 25.53 ± 2.84 mm. No difference was detected between 3D MRI PD‐SPACE‐COR imaging and cadaveric measurement (*p*_overall_ = 0.78), regardless of genders (*p*_Male_ = 0.63, *p*_Female_ = 0.87). Moreover, gender difference has been found (*p*_3D MRI PD‐SPACE‐COR_ = 0.003, *p*_Cadaveric morphometry_ = 0.01).

**TABLE 2:** The distance between safety margin and coracoid tip measured by 3D MRI PD‐SPCAE‐COR and cadaveric mophometry

Group	3D MRI PD‐SPACE‐COR (n = 24)	Cadaveric morphometry (n = 32)	*p*‐value
Overall (mm)	24.28 ± 2.17 (20.10–29.10)	25.53 ± 2.84 (20.70–32.02)	0.78
Male (mm)	25.06 ± 1.92 (21.50–29.10)	26.63 ± 2.80 (22.33–32.02)	0.63
Female (mm)	22.38 ± 1.51 (20.10–24.10)	24.11 ± 2.29 (20.70–27.11)	0.87

*Note*: The data were expressed as mean ± standard division (range).

## Discussion

In this study, the 3D MRI PD‐SPACE‐COR protocol was of a high sensitivity in CCL attachment imaging and had good accuracy in preoperatively evaluating the SM for coracoid process transfer. The inconsistent SMs between the Chinese cadaveric cohort and previous reports as well as gender difference, in turn, indicate that necessity of preoperatively geometrical planning in Latarjet shoulder reconstruction using the 3D MRI PD‐SPACE‐COR protocol.

### The Superiority of PD‐SPACE‐COR in Predicting Safety Margin

The PD‐SPACE‐COR was better than PD‐SPACE‐FS‐COR in the clarity and sensitivity of CCLs indicated by a higher ranking, significant statistic by McNemar test, and moderate disagreement by Cohen's kappa analysis.^14^ The inconsistency between PD‐SPACE‐COR and PD‐SPACE‐FS‐COR may be caused by the fat suppression technology. Because the adipose tissue has been commonly found where tendon or ligament inserts bone.^15^ The fat‐suppressed protocol may have screened the adipose signal,^16^ which decreases the clarity of the CCLs in PD‐SPACE‐FS‐COR imaging. Additionally, the motion artifacts were supposed to be the cause of the only PD‐SPACE‐COR negative case (95.8% sensitivity in 24 cases).

### The Feasibility of PD‐SPACE‐COR in Predicting Safety Margin

Iteratively denoised 3D SPACE allows retrospective MPRs of the osseous structures, for example, female pelvis.^17^ Moreover, the 3D SPACE T2‐weighted sequence has a good diagnostic quality in evaluating cervical and lumbar spine MRI anatomy.^18^, ^19^ In terms of ligament imaging, 3D SPACE precedes 2D PD‐weighted sequences in visualizing wrist ligaments.^20^ The controlled aliasing in parallel imaging results in higher acceleration (CAIPIRINHA) accelerated SPACE enables fast isotropic 3D TSE MRI of the ankle, optimizing visualization of the curved and oblique ligaments and tendons.^21^ But, the specificity of 3D CAIPIRINHA SPACE TSE was 80% in a previous report in assessing acromioclavicular joint abnormalities, whose manifestations include the periarticular ligament abnormal thickening and high signal intensity of these ligaments.^22^ The MRI contrast is determined by the magnetic properties and number of hydrogen nuclei in the studied region. The results of imaging can be optimized by selecting sequences with different weightings. PD‐weighted imaging does not display the magnetic characteristics of the hydrogen nuclei but the number of nuclei in the targeted area. Hence, it has been proven in this study that the 3D PD‐weighted SPACE imaging has a sensitivity as high as 95.8% in shaping the osteoligamentous structures that is, the coracoid process and the attachments of the CCLs in a narrow space, and determining SM. Ultimately, PD‐SPACE‐COR may be applied to assess the attachment of periarticular tissues of similar properties to CCLs in other orthopedic surgeries.

### The Reliability of PD‐SPACE‐COR in Predicting Safety Margin

Similar to our finding, the reliability of the 3D MRI in imaging other bony and ligamentous structures has been revealed.^23^, ^24^, ^25^ The 3D MRI predicted glenoid bone defect and showed no difference with anatomically determined values (for 10% defect, *P* = 0.27; for 25% defect, *P* = 0.73).^24^ A difference of less than 6% has been reported between the cadaveric data and 3D MR imaging with a strongly correlation (*r* ^2^ = 0.92) in determining glenoid bone loss.^23^ Moreover, in terms of imaging tiny structures, for example, the CCL attachments, 3D MRI is of high priority. In as narrow a space as intercarpal joints, anatomy on the radial side of the scaphoid inter‐ligamentous connections confirmed the same ligament configuration as 3D MRI scanning.^25^ Hence, in this study, the reliability of the 3D MRI PD‐weighted SPACE sequence in imaging the CCL attachments has been verified using with tactile measurement. However, a rigorous way to determine the reliability of 3D MRI PD‐weighted SPACE sequence in predicting SM, for example, a cohort study focusing the postoperative outcomes of Lartajet shoulder reconstruction with preoperatively PD‐SPACE‐COR evaluation, is advocated for further investigation.

### Preoperative Safety Margin Evaluation Serves Personalized Medicine

Personalized management and precision medicine in bone and mineral disorders and bone tissue engineering have been proposed in disease state and traumatic condition.^26^, ^27^ Population differences have been revealed: the SM (25.53 ± 2.84 mm) detected in the Chinese group in this study was similar to that in the Japanese group (24.8 ± 3.4 mm), higher than the data from the Mongolian population (23.93 ± 2.32 mm), while lower than the American (28.5 ± 5.1 mm) and Brazilian (2.79 ± 0.33 cm).^5^, ^28^, ^29^, ^30^ Moreover, the gender difference was detected in this study which is similar to the findings in previous reports.^31^ In an American anatomical research with no explicit mentioning of the cadaver population,^31^ a similar average SM (25.1 mm, range 22.1–28.1) is reported while the SM in males (28.1 mm, range 25.1–31.2) is higher and that in females (22.0 mm, range 18.2–25.9) is lower compared to the Chinese population. In terms of the screw used to secure the coracoid graft to the anterior glenoid, two screws of ΦO 3.5–4.5 mm corresponds to a coracoid graft length of 20–25 mm,^32^, ^33^, ^34^ which has been widely recognized in Lartarjet surgery. However, one screw has been applied in patients with insufficient SM and poor drill hole positioning,^35^, ^36^ or to prevent insufficient external bone bridge in the condition of SM being too short.^30^ Totally, a series of variates, including: (i) The glenoid defect size; (ii) population difference; (iii) gender difference; and (iv) The minimum length required for screw insertion, for example, the screw size and the screw number,^30^, ^32^, ^33^, ^34^ take part in determining the surgical outcomes of Latarjet shoulder reconstruction, which emphasize the essential of preoperatively predicting SM in coracoid process transfer in the climate of personalized medicine.

### Strengths and Limitations

In this study, 3D MRI PD‐SPACE‐COR was qualified in predicting the SM of the coracoid process for osteotomy in Latarjet shoulder reconstruction due to its high accuracy, compared with conventional MRI and 3D CT. In a previous report, conventional MRI is limited by high slice thicknesses and large inter‐slice interval,^37^ which results in the absent CCL attachments in conventional MR images (Fig. S2). Moreover, 3D CT has been employed in the general morphometric analysis of coracoid process rather than determining the bony landmark for osteotomy.^38^, ^39^ Though CT is of a relative low clarity and resolution compared to MRI, it takes a longer acquisition time for the 3D sequence (VIBE sequence acquisition time on average was 4: 16).^10^ Therefore, CT is of high priority in clinics, which leads to the limitations of this study: (i) the 3D MRI data corresponding specimens is not assessable from the medical records, which causes a risk of bias in drawing conclusions; and (ii) indeed, the time cost and expenditure of 3D MRI restrict the size of the imaging cohort.

### Conclusions

This study suggests that 3D MRI PD‐SPACE‐COR is qualified for preoperatively predicting SM in coracoid process transfer, which optimizes the surgical decision and may motivate the development of personalized medicine in the Latarjet shoulder reconstruction surgery.

## Authors Contributions

Conceptualization, ZR and TY; methodology, ZR, HP and JM; data curation, ZR and HP; writing—original draft preparation, ZR and XH; writing—review and editing, ZR, XH and TY; supervision, TY and YZ; funding acquisition, TY.

## Conflicts of Interest

The authors declare no conflict of interest.

## Ethical Approval

This study was performed following the principles of Declaration of Helsinki. Ethical approval was granted by The Affiliated Hospital of Qingdao University (Date: Dec, 3rd, 2020. IRB approval No. QYFYWZLL26070).

## Supporting information


**Fig. S1.** Specimen preparation in cadaveric morphometric analysis. (A). Soft tissues, including skin, subcutaneous tissues, deltoid, and pectoralis major muscle, are removed to expose the coracoid process, conjoint tendon (CT), pectoralis minor (PM), coracoacromial ligament (CAL), and coracoclavicular ligament [CCL, including trapezoid ligament (TL) and conoid ligament (CL)]. (B). The root of the coracoid process is sawed off, ligaments and tendons were removed from the coracoid process.

**Fig. S2.** The images by conventional MRI (FSE‐T2WI). The attachments of the coracoclavicular ligament (CCL) was not observed in the coronal (A, B, C), sagittal (D, E, F), horizontal (G, H, I) planes with 3.0 mm slice thickness, 0.3 mm slice gap, 256 × 256 matrix and 20 field‐of‐view.

## References

[1] Joshi D , Gupta L , Tanwar M , et al. Anthropometric and radiologic measurements of coracoid dimensions and clinical implications in an Indian population. Orthop J Sports Med. 2018;6(3):2325967118761635. 10.1177/2325967118761635 29619396 PMC5871059

[2] Rossi L , Tanoira I , De Cicco F , et al. Traditional versus congruent‐arc Latarjet anatomic and biomechanical perspective. EFORT Open Rev. 2021;6(4):280–7. 10.1302/2058-5241.6.200074 34040805 PMC8142695

[3] Armitage M , Elkinson I , Giles J , et al. An anatomic, computed tomographic assessment of the coracoid process with special reference to the congruent‐arc latarjet procedure. Arthroscopy. 2011;27(11):1485–9. 10.1016/j.arthro.2011.06.020 21924857

[4] Hurley E , Jamal M , Ali Z , et al. Long‐term outcomes of the Latarjet procedure for anterior shoulder instability: a systematic review of studies at 10‐year follow‐up. J Shoulder Elbow Surg. 2019;28(2):e33–9. 10.1016/j.jse.2018.08.028 30545784

[5] Shibata T , Izaki T , Miyake S , Doi N , Arashiro Y , Shibata Y , et al. Predictors of safety margin for coracoid transfer: a cadaveric morphometric analysis. J Orthop Surg. 2019;14(1):174. 10.1186/s13018-019-1212-z PMC6558900 31182130

[6] Mogharrabi B , Cabrera A , Chhabra A . 3D isotropic spine echo MR imaging of elbow: how it helps surgical decisions. Eur J Radiol Open. 2022;9:100410. 10.1016/j.ejro.2022.100410 35281319 PMC8904410

[7] Endler CH, Faron A, Isaak A , et al. Fast 3D isotropic proton density‐weighted fat‐saturated MRI of the KNEe at 1.5 T with compressed sensing: comparison with conventional multiplanar 2D sequences. 2021;193:813–821. 10.1055/a-1337-3351 33535259

[8] Xin L , Luo J , Chen M , et al. Anatomy and correlation of the coracoid process and Coracoclavicular ligament based on three‐dimensional computed tomography reconstruction and magnetic resonance imaging. Med.Sci.Monit. 2021;27:e930435–1. 33947821 10.12659/MSM.930435 PMC8080654

[9] Paul RW , DeBernardis DA , Hameed D , Clements A , Kamel SI , Freedman KB , et al. Effect of preoperative MRI coracoid dimensions on postoperative outcomes of Latarjet treatment for anterior shoulder instability. Orthop J Sports Med. 2022;10(7):23259671221083967. 35923867 10.1177/23259671221083967 PMC9340370

[10] Stillwater L , Koenig J , Maycher B , Davidson M . 3D‐MR vs. 3D‐CT of the shoulder in patients with glenohumeral instability. Skeletal Radiol. 2017;46(3):325–31. 10.1007/s00256-016-2559-4 28028575

[11] Gyftopoulos S , Beltran L , Yemin A , et al. Use of 3D MR reconstructions in the evaluation of glenoid bone loss: a clinical study. Skeletal Radiol. 2014;43(2):213–8. 10.1007/s00256-013-1774-5 24318071

[12] Gyftopoulos S , Yemin A , Mulholland T , Bloom M , Storey P , Geppert C , et al. 3DMR osseous reconstructions of the shoulder using a gradient‐echo based two‐point Dixon reconstruction: a feasibility study. Skeletal Radiol. 2013;42(3):347–52. 10.1007/s00256-012-1489-z 22829026

[13] Kloth J , Winterstein M , Akbar M , et al. Comparison of 3D turbo spin‐echo SPACE sequences with conventional 2D MRI sequences to assess the shoulder joint. Eur J Radiol. 2014;83(10):1843–9. 10.1016/j.ejrad.2014.06.011 25082477

[14] Landis J , Koch G . The measurement of observer agreement for categorical data. Biometrics. 1977;33(1):159–74. 10.2307/2529310 843571

[15] Benjamin M , Redman S , Milz S , Büttner A , Amin A , Moriggl B , et al. Adipose tissue at entheses: the rheumatological implications of its distribution. A potential site of pain and stress dissipation? Ann Rheum Dis. 2004;63(12):1549–55. 10.1136/ard.2003.019182 15547077 PMC1754852

[16] Mao J , Yan H , Brey W , et al. Fat tissue and fat suppression. Magn Reson Imaging. 1993;11(3):385–93. 10.1016/0730-725x(93)90071-k 8505872

[17] Hausmann D , Pindur A , Todorski I , Weiland E , Kuehn B , Zhou K , et al. Quantitative assessment of iteratively denoised 3D SPACE with inner‐volume excitation and simultaneous multi‐slice BLADE for optimizing female pelvis magnetic resonance imaging at 1.5 T. Acad Radiol. 2022;12:2370. 10.1016/j.acra.2022.06.015 35871059

[18] Chokshi F , Sadigh G , Carpenter W , et al. Diagnostic quality of 3D T2‐SPACE compared with T2‐FSE in the evaluation of cervical spine MRI anatomy. Am J Neuroradiol. 2017;38(4):846–50. 28154126 10.3174/ajnr.A5080 PMC7960240

[19] Hossein J , Fariborz F , Mehrnaz R , Babak R . Evaluation of diagnostic value and T2‐weighted three‐dimensional isotropic turbo spin‐echo (3D‐SPACE) image quality in comparison with T2‐weighted two‐dimensional turbo spin‐echo (2D‐TSE) sequences in lumbar spine MR imaging. Eur J Radiol Open. 2019;6:36–41. 30619918 10.1016/j.ejro.2018.12.003 PMC6312863

[20] Götestrand S , Björkman A , Björkman‐Burtscher IM , Ab‐Fawaz R , Kristiansson I , Lundin B , et al. Visualization of wrist ligaments with 3D and 2D magnetic resonance imaging at 3 tesla. Acta Radiol. 2022;63(3):368–75. 33657847 10.1177/0284185121994044

[21] Kalia V , Fritz B , Johnson R , Gilson WD , Raithel E , Fritz J . CAIPIRINHA accelerated SPACE enables 10‐min isotropic 3D TSE MRI of the ankle for optimized visualization of curved and oblique ligaments and tendons. Eur Radiol. 2017;27:3652–61. 28116515 10.1007/s00330-017-4734-y

[22] Hou B , Li Y , Xiong Y , Morelli JN , Wang J , Liu C , et al. Comparison of CAIPIRINHA‐accelerated 3D fat‐saturated‐SPACE MRI with 2D MRI sequences for the assessment of shoulder pathology. Eur Radiol. 2022;32:593–601. 34258637 10.1007/s00330-021-08183-3

[23] Huijsmans P , Haen P , Kidd M , et al. Quantification of a glenoid defect with three‐dimensional computed tomography and magnetic resonance imaging: a cadaveric study. J Shoulder Elbow Surg. 2007;16(6):803–9. 10.1016/j.jse.2007.02.115 18061117

[24] Yanke A , Shin J , Pearson I , et al. Three‐dimensional magnetic resonance imaging quantification of glenoid bone loss is equivalent to 3‐dimensional computed tomography quantification: cadaveric study. Arthroscopy. 2017;33(4):709–15. 10.1016/j.arthro.2016.08.025 27923707

[25] Mania S , Boudabbous S , Delattre B , et al. Anatomical and radiological description of ligament insertions on the radial aspect of the scaphoid bone. Hand Surg Rehabil. 2022;41(4):445–51. 10.1016/j.hansur.2022.05.008 35660467

[26] Zhao B , Peng Q , Zhou R , et al. Precision medicine in tissue engineering on bone. Methods Mol Biol. 2020;2204:207–15. 10.1007/978-1-0716-0904-0_18 32710327

[27] Jovanovich A , Kendrick J . Personalized Management of Bone and Mineral Disorders and precision medicine in end‐stage kidney disease. Semin Nephrol. 2018;38(4):397–409. 10.1016/j.semnephrol.2018.05.009 30082059 PMC6615060

[28] Lian J , Dong L , Zhao Y , Sun J , Zhang W , Gao C . Anatomical study of the coracoid process in Mongolian male cadavers using the Latarjet procedure. J Orthop Surg Res. 2016;11(1):126. 10.1186/s13018-016-0461-3 27776520 PMC5078878

[29] Dolan C , Hariri S , Hart N , et al. An anatomic study of the coracoid process as it relates to bone transfer procedures. J Shoulder Elbow Surg. 2011;20(3):497–501. 10.1016/j.jse.2010.08.015 21106399

[30] Terra BB , Ejnisman B , de Figueiredo EA , Cohen C , Monteiro GC , de Castro Pochini A , et al. Anatomic study of the coracoid process: safety margin and practical implications. Arthroscopy. 2013;29(1):25–30. 10.1016/j.arthro.2012.06.022 23183115

[31] Chahla J , Marchetti D , Moatshe G , et al. Quantitative assessment of the coracoacromial and the coracoclavicular ligaments with 3‐dimensional mapping of the coracoid process anatomy: a cadaveric study of surgically relevant structures. Arthroscopy. 2018;34(5):1403–11. 10.1016/j.arthro.2017.11.033 29395551

[32] Imai S . A new guide for the arthroscopically assisted Latarjet procedure. JBJS Open Access. 2021;6(1):e20.00141–9. 10.2106/jbjs.Oa.20.00141 PMC7963512 33748647

[33] Weppe F , Magnussen R , Lustig S , Demey G , Neyret P , Servien E . A biomechanical evaluation of bicortical metal screw fixation versus absorbable interference screw fixation after coracoid transfer for anterior shoulder instability. Arthroscopy. 2011;27(10):1358–63. 10.1016/j.arthro.2011.03.074 21703807

[34] Willemot L , Wodicka R , Bosworth A , et al. Influence of screw type and length on fixation of anterior glenoid bone grafts. Shoulder Elbow. 2018;10(1):32–9. 10.1177/1758573217704817 29276535 PMC5734526

[35] Omae H . CT image evaluation of one‐screw fixation in the Latarjet procedure. Trauma Case Rep. 2020;30:100372. 10.1016/j.tcr.2020.100372 33204803 PMC7649353

[36] Athwal G , Meislin R , Getz C , et al. Short‐term complications of the arthroscopic Latarjet procedure: a North American experience. Arthroscopy. 2016;32(10):1965–70. 10.1016/j.arthro.2016.02.022 27160460

[37] Lee Y , Hahn S , Lim D , et al. Articular cartilage grading of the knee: diagnostic performance of fat‐suppressed 3D volume isotropic turbo spin‐echo acquisition (VISTA) compared with 3D T1 high‐resolution isovolumetric examination (THRIVE). Acta Radiol. 2017;58(2):190–6. 10.1177/0284185116646142 27207633

[38] Jia Y , He N , Liu J , Zhang G , Zhou J , Wu D , et al. Morphometric analysis of the coracoid process and glenoid width: a 3D‐CT study. J Orthop Surg. 2020;15(1):69. 10.1186/s13018-020-01600-1 PMC7038565 32093704

[39] Imma I , Nizlan N , Ezamin A , et al. Coracoid process morphology using 3D‐CT imaging in a Malaysian population. Malays Orthop J. 2017;11(2):30–5. 10.5704/moj.1707.012 29021876 PMC5630048

