# Supplementary material for: 3D MRI PD‐SPACE‐COR Predicting Safety Margin for Coracoid Transfer
Source: Orthop Surg. 2023 Apr 24;15(6):1514–20. doi: 10.1111/os.13719 (PMC10235170; doi:10.1111/os.13719)
Supplement: Supplementary file 1 — Fig. S1. Specimen preparation in cadaveric morphometric analysis. (A). Soft tissues, including skin, subcutaneous tissues, deltoid, and pectoralis major muscle, are removed to expose the coracoid process, conjoint tendon (CT), pectoralis minor (PM), coracoacromial ligament (CAL), and coracoclavicular ligament [CCL, including trapezoid ligament (TL) and conoid ligament (CL)]. (B). The root of the coracoid process is sawed off, ligaments and tendons were removed from the coracoid process. [file OS-15-1514-s001.docx]

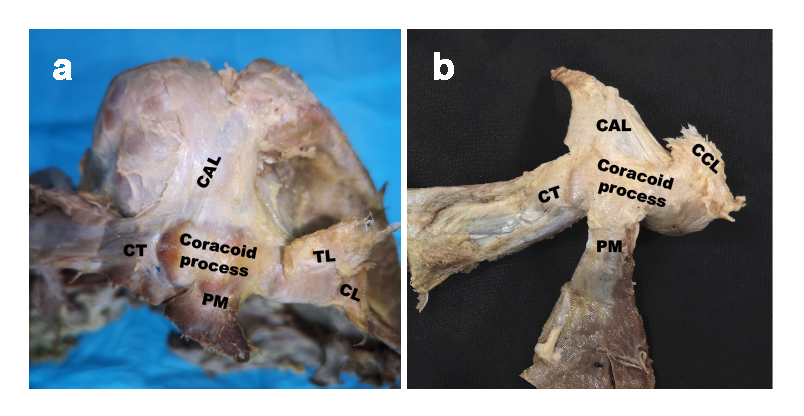


**Supplemental Fig. 1.** Specimen preparation in cadaveric morphometric analysis. a). Soft tissues, including skin, subcutaneous tissues, deltoid, and pectoralis major muscle, are removed to expose the coracoid process, conjoint tendon (CT), pectoralis minor (PM), coracoacromial ligament (CAL), and coracoclavicular ligament [CCL, including trapezoid ligament (TL) and conoid ligament (CL)]. b). The root of the coracoid process is sawed off, ligaments and tendons were removed from the coracoid process.
