# Supplementary material for: 3D MRI PD‐SPACE‐COR Predicting Safety Margin for Coracoid Transfer
Source: Orthop Surg. 2023 Apr 24;15(6):1514–20. doi: 10.1111/os.13719 (PMC10235170; doi:10.1111/os.13719)
Supplement: Supplementary file 2 — Fig. S2. The images by conventional MRI (FSE‐T2WI). The attachments of the coracoclavicular ligament (CCL) was not observed in the coronal (A, B, C), sagittal (D, E, F), horizontal (G, H, I) planes with 3.0 mm slice thickness, 0.3 mm slice gap, 256 × 256 matrix and 20 field‐of‐view. [file OS-15-1514-s002.docx]

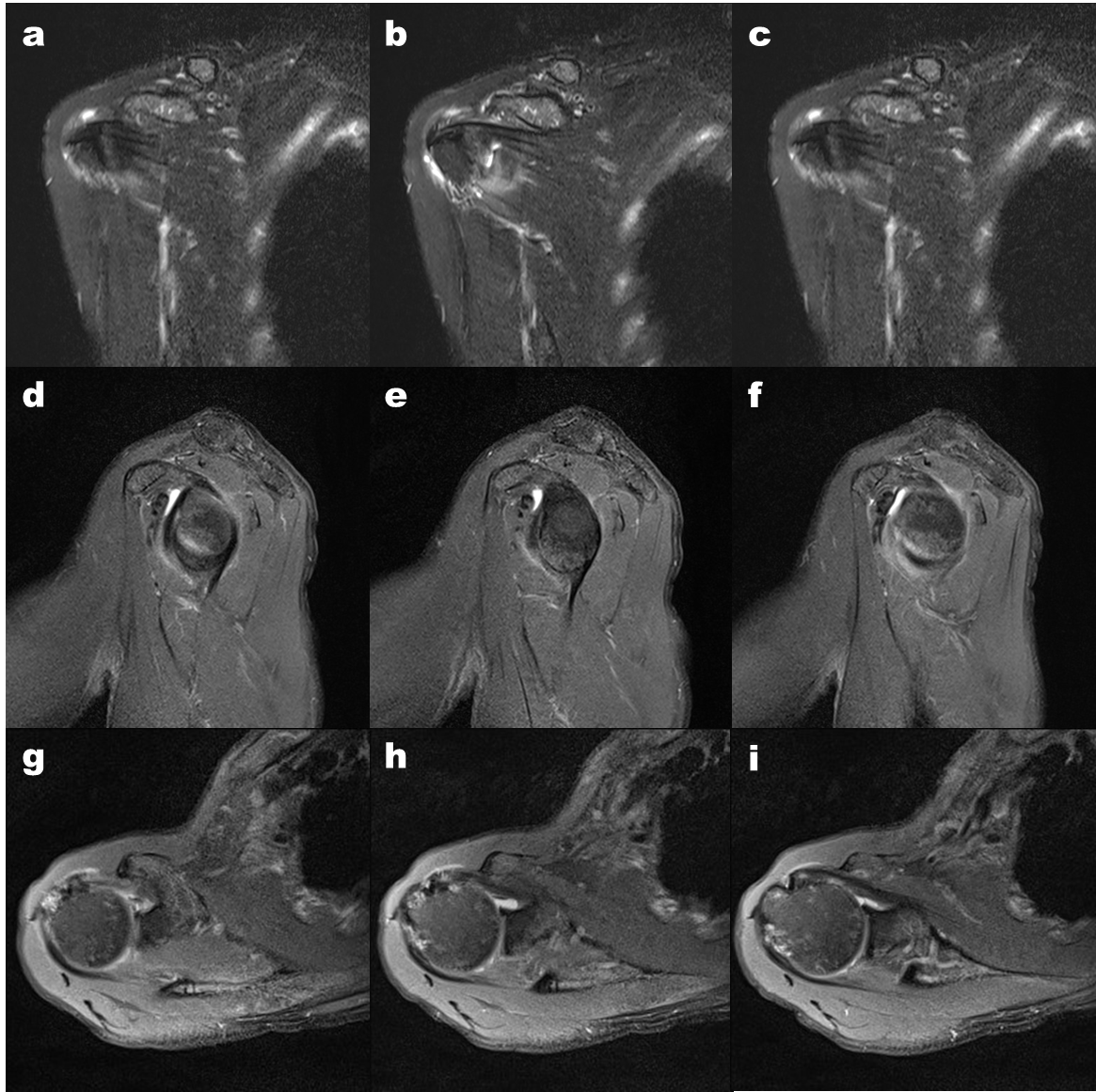


**Supplemental Fig. 2.** The images by conventional MRI (FSE-T2WI). The attachments of the coracoclavicular ligament (CCL) was not observed in the coronal (a, b, c), sagittal (d, e, f), horizontal (g, h, i) planes with 3.0 mm slice thickness, 0.3 mm slice gap, 256 × 256 matrix and 20 field-of-view.
